# Impact of hands-on workshops on future medical students' motivation, confidence, and career aspirations: an observational study

**DOI:** 10.25122/jml-2025-0030

**Published:** 2025-02

**Authors:** Dumitru Sutoi, Alexandru Cristian Cindrea, Daian Ionel Popa, Cosmin Iosif Trebuian, Carmen Williams, Maria Sutoi, Alexandru Bogdan Puscas, Adina Maria Marza, Florina Buleu, Bogdan Chiu, George Marin, Vlad Mulcutan Chis, Anda Ciontos, Luca Darie Sabau, Ovidiu Alexandru Mederle

**Affiliations:** 1 Department of Surgery, Emergency Discipline, Victor Babes University of Medicine and Pharmacy, Timisoara, Romania; 2 Emergency Clinical Municipal Hospital, Timisoara, Romania; 3 Doctoral School, Faculty of General Medicine, Victor Babes University of Medicine and Pharmacy, Timisoara, Romania; 4 Emergency County Hospital, Reșita, Romania; 5 Pius Brinzeu Emergency Clinical County Hospital, Timisoara, Romania; 6 Department of Cardiology, Victor Babes University of Medicine and Pharmacy, Timisoara, Romania; 7 Victor Babes University of Medicine and Pharmacy, Timisoara, Romania

**Keywords:** Medical education, workshop, motivation, self-confidence, practice, pre-medical, teaching

## Abstract

Workshops serve as an extrinsic motivational tool for medical students, enhancing their knowledge, self-confidence, and practical skills. These workshops could have a similar impact on future medical students, who may have various reasons for their aspiration to follow a career in medicine. The main goal of our study was to demonstrate that participating in hands-on workshops boosts the motivation of future medical students to work toward a career in medicine. The second goal was to evaluate the amount of first aid assistance participants provided, their willingness to deliver first aid, and the impact on self-perceived confidence in their practical skills after attending this experience. Workshops were conducted across multiple cities, engaging 540 participants between March and November 2024. At year-end, a custom questionnaire assessed their motivation, self-confidence, and medical career aspirations. A total of 186 participants met the inclusion criteria and were analyzed. Participants had a median age of 18.5 years (18–19.75) and graduated high school (55.4%). Most chose to follow medicine due to a strong desire to help or an exclusive desire to practice in this field. Significant increases in respondents' Likert scale ratings were observed before and after the workshops (*P* < 0.001, Wilcoxon signed-rank test). No significant differences were found when comparing responses between different workshops. In conclusion, the results of the motivating factors align with the trends in other high-income countries. Also, workshops serve as an extrinsic motivational source, increasing students’ self-confidence and theoretical and practical knowledge.

## INTRODUCTION

The most important factors that influence future medical students' decisions to pursue a career in medicine include humanitarian reasons, such as a desire to help others [1,2]; societal factors (such as prestige and reputation); and scientific reasons, such as interest in medicine [3]. These motivational factors vary across countries, often influenced by a nation's income level. Romania is recognized as a high-income country [4]. In other high-income nations with a similar geographical position, such as Hungary and Poland, the previously mentioned motivational factors were the most significant [5,6].

For young people who desire to become a physician, understanding the true nature of the profession is essential when following a career in medicine. Scientific factors also play a crucial role in motivating future medical students [7]. Opportunities such as participation in hands-on workshops provide valuable visual and practical experience, helping them better understand medicine. Additionally, these workshops enhance learning and boost confidence, serving as an effective extrinsic motivational tool [8].

Extrinsic motivational sources are essential for determining and stimulating future and actual students in the learning process. As Cook *et al*. [9] summarize, the five contemporary motivational theories—expectancy-value, social-cognitive, attribution, goal orientation, and self-determination—illustrate that a hands-on workshop can inspire prospective medical students by addressing each of these motivational frameworks.

Despite its importance, motivation in medical education is not well-researched [10]. In Romania, admission to medical schools relies on a theoretical knowledge-based exam, which primarily evaluates students' memorization skills. This approach may negatively impact their motivation. Integrating hands-on workshops in medicine preparation not only serves as an extrinsic motivational source but also develops intrinsic motivation by reducing stress and enhancing self-confidence [11].

The literature presents that workshops bring advantages to students’ preparation, such as developing interest and understanding of academic medicine [12], improving patients’ safety and increasing the quality of medical acts [13], and developing clinical skills [14,15]. Moreover, workshops significantly improve first-year medical students’ understanding of medical topics, positively impacting their learning [16].

These advantages can be brought early, specifically before medical school admission, to prepare future medical students. Also, the benefits brought to society by teaching the general population the rules of first aid represent the basic principle that can complement the civic sense of people who are eager to intervene in critical situations but lack the necessary knowledge. When it comes to emergencies, the moral courage of citizens should become a principle, an unwritten rule, and not just a simple quality that only some people possess, optionally [17].

Unfortunately, in Romania, many future medical students have limited opportunities to participate in workshops that could boost motivation, knowledge, and practical experience that can improve their preparation for medical school.

The current study can solve the problem of offering opportunities, such as hands-on workshops, for future medical students. These workshops positively impact their learning process by providing valuable experience correlating theoretical knowledge with practical skills while boosting their desire to study medicine, motivation, self-confidence, and the courage to provide first aid in emergencies.

The primary objective of our study was to prove that participation in hands-on workshops enhances the motivation of future medical students to pursue a career in medicine. The second aim of this article was to assess the extent of first aid assistance participants have provided, their willingness to offer first aid in the future, and their self-reported confidence in their practical skills following a series of workshops.

## MATERIAL AND METHODS

An observational study was conducted in December 2024 involving participants who attended a series of events titled “Learn by Practice”. These events took place over multiple sessions between March and November 2024. To assess the impact of hands-on workshops on individuals aspiring to pursue medical studies, all participants were administered a custom-designed survey.

**Figure 1:**
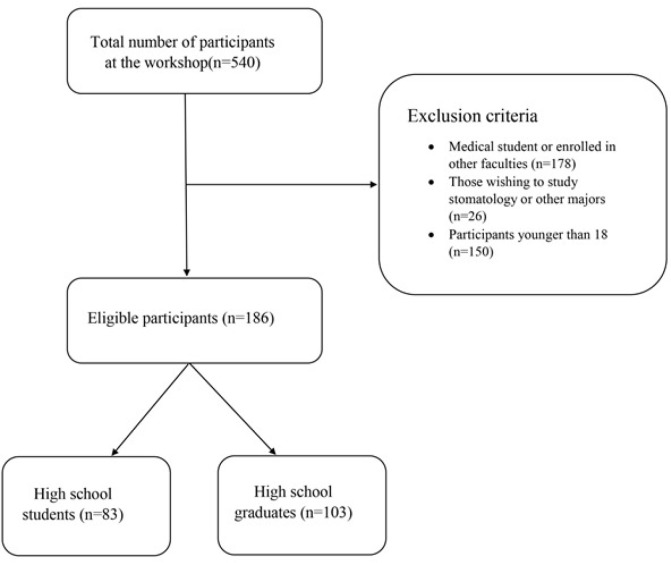
Study flowchart

A total of 540 participants attended the events. The inclusion criteria for the study were individuals not already enrolled as medical students, those aspiring to pursue general medicine, individuals aged 18 years or older, and those who provided informed consent to participate. Exclusion criteria included participants who were already students (including those in medical school), individuals intending to study dental medicine, nursing, or other fields, and those under 18. The study flowchart is presented in Figure 1.

### Workshop structure

“Learn by Practice” is a program created for medical students and young people from the general population who aspire to pursue a medical career. The topics covered in the workshops generally consist of common maneuvers that allow participants to gain practical experience, which they can then correlate to their theoretical knowledge, offering them an opportunity to uncover the reality of medical life. This event included five different medical workshops:

- Become a Hero (emergency medicine and first aid field): with main practical topics: ABC evaluation, BLS (basic life support), lateral safety position, Heimlich maneuver, CPR (cardiopulmonary resuscitation), and basic leadership notions.
- Needle skills (nursing and interdisciplinary field): these included venous blood collection, intramuscular, intradermal, and subcutaneous injection, peripheral venous catheter insertion, notions of asepsis, and antisepsis.
- Basic Skills in Surgery and Surgical Approach in the Emergency Room (surgery field): notions of asepsis and antisepsis, surgical tools, basic knots, and sutures.
- Heal the Wound (nursing and first aid field): wound management and debridement, wound dressing, tetanus vaccine administration, hemostasis using an improvised tourniquet, and stasis by pressure.
- First Aid Save the Trauma (non-technical skills, emergency medicine, and first aid field): leadership abilities, teamwork abilities, coordination of the rescue team, the ability to react and interact with the patient, basic ABCDE evaluation, fitting the cervical collar, peripheral venous catheter insertion, dressing of a wound, hemostasis.

An accredited trainer led the events. The workshops were taught by either specialized physicians or final-year medical students.

### Data collection and questionnaire design

Data was collected using the Google Forms Platform for participants from Constanta and Oradea (66) and the same printed version for participants from Timisoara, Resita, and Arad (120).

Our research instrument was a self-developed questionnaire, as no validated Romanian questionnaire assesses parameters related to the motivation to pursue medicine, self-confidence, and other aspects specific to our workshops. The questionnaire consisted of 33 items grouped into three sections: (1) demographic information, (2) workshop participation and knowledge acquisition, and (3) personal perceptions. It assessed five key dimensions: motivation to pursue medicine, self-confidence, perceived impact of the workshops, changes in theoretical and practical knowledge, and willingness to apply first-aid skills.

Participants completed the questionnaire anonymously, either online via a secure survey link or in person, using preprinted forms distributed by the workshop organizers between December 15–31, 2024. Higher scores on Likert scale questions (ranging from 1 to 10) indicated increased motivation, confidence, or knowledge acquisition. Sample items include: 'On a scale from 1 to 10, how confident were you in your first-aid skills before the workshop?' and 'What motivated you to attend the Learn by Practice workshop?' The full questionnaire can be found in Supplementary Material M1.

Supplementary Material

### Statistical analysis

Descriptive statistics consist of median and interquartile range (IQR) for continuous variables and absolute counts and percentages for categorical variables. Skewness, kurtosis, and Shapiro–Wilk tests were used to assess the distribution of variables. Due to the abnormal data distribution, either the Kruskal–Wallis or Mann–Whitney U tests were used to compare the data distribution between groups. When the Kruskal–Wallis was initially applied, pairwise comparisons using the Mann–Whitney U test were conducted to explore potential subtle differences between the groups. Comparisons between paired before-and-after data were conducted utilizing the Wilcoxon signed-rank test. Cronbach’s alpha was the method of choice for assessing internal reliability. Data analysis was conducted utilizing the R software v4.4.1. Packages 'dplyr', 'effsize', 'flextable', 'ggplot2', 'moments', 'officer', 'purrr', 'psych', 'stringr', 'tidyverse' and 'viridis' and Microsoft Excel Worksheets 2016 were used.

## RESULTS

The final database comprised 186 responses. Of these, 103 (55.38%) had already graduated from high school, while 83 (44.62%) were still pupils. The median age was 18.5 years, with an interquartile range (IQR) of 18–19.75 years (Table 1). To assess internal consistency, Cronbach’s alpha was calculated separately for the pre-intervention and post-intervention items. The analysis yielded acceptable reliability values for both sets of questions, with a Cronbach’s alpha of 0.775 for the pre-intervention items and 0.754 for the post-intervention items, indicating good internal consistency.

**Table 1:** Descriptive statistics

Variable		Median (IQR)	Skewness	Kurtosis	*P* value^*^
Age		18.5 (18 - 19.75)	2.19	8.22	<0.001*
Involvement in the learning process	Before	7 (6 - 8)	-0.13	2.87	<0.001*
	After	9 (9 - 10)	-0.78	2.78	<0.001*
Motivation to learn more		10 (7 - 10)	-1.21	2.98	<0.001*
Motivation	Before	8 (7 - 9)	-1.12	4.71	<0.001*
	After	10 (10 - 10)	-2.17	7.33	<0.001*
Increased motivation to learn		10 (8 - 10)	-1.25	3.00	<0.001*
Self-confidence	Before	7 (5 - 8)	-0.19	2.57	<0.001*
	After	8 (7 - 9)	-0.40	2.43	<0.001*
Self-trust		10 (8 - 10)	-1.28	2.93	<0.001*
Theoretical knowledge	Before	6 (4 - 7)	-0.11	2.69	<0.001*
	After	10 (9 - 10)	-0.83	2.59	<0.001*
Practical abilities	Before	5 (3 - 6)	0.25	2.36	<0.001*
	After	10 (9 - 10)	-0.89	2.54	<0.001*
Weekly number of hours spent studying	Before	10 (6 - 15)	0.16	2.13	<0.001*
	After	14 (10 - 18)	-0.25	1.95	<0.001*
Correlation between practical and theory	Before	6 (5 - 7)	-0.04	2.69	<0.001*
	After	9 (8 - 10)	-0.76	2.59	<0.001*
Ability to administer first aid	Before	4 (2 - 6)	0.58	2.61	<0.001*
	After	8 (6 - 9)	-0.97	3.24	<0.001*
Would you administer first aid now?		10 (9 - 10)	-2.62	10.95	<0.001*
Do you think first aid should be taught more in schools?		10 (7 - 10)	-1.20	2.70	<0.001*
Is the workshop a source of extrinsic motivation?		10 (7 - 10)	-1.24	3.06	<0.001*
Did the workshop increase my determination to learn more?		10 (8 - 10)	-1.33	3.18	<0.001*
I feel like I am preparing to become a doctor		10 (9 - 10)	-1.62	4.01	<0.001*

* Statistically significant, P < 0.01, Shapiro–Wilk test

**Figure 2:**
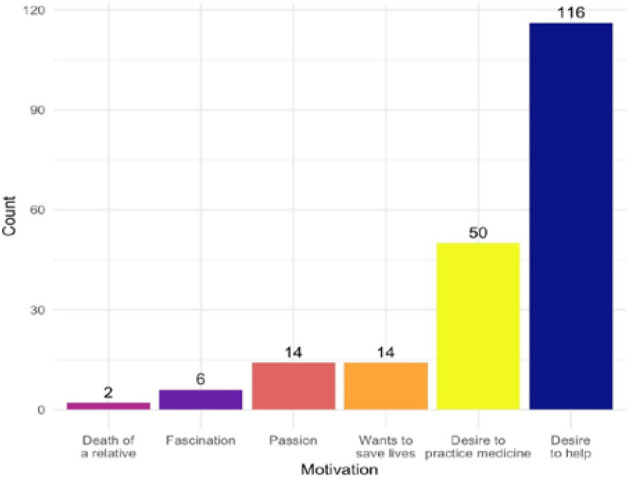
Column graph representing respondent’s main motivations to follow medical studies

Figure 2 showcases the motivation of the participants to study medicine in the future. The desire to help was, by far, the main drive of the participants. Responses with less than 1 appearance were not depicted. Other answers include: “It is all I wanted since I was little”, “I cannot see myself doing anything else”, “I have enjoyed studying biology and chemistry”, “I see it as a noble profession”, “I want to have a significant contribution upon our society”, “I want to innovate, to find new cures”, “to heal both souls and bodies”.

The descriptive statistics for the data assessed utilizing the Likert-type scale are summarized in Table 1. For all the parameters measured before and after the workshop, the difference between the values was statistically significant (with *P* values <0.01, Wilcoxon signed-rank test). The most significant increase observed was in self-confidence regarding practical abilities, with the median rising from 5 to 10.

**Figure 3:**
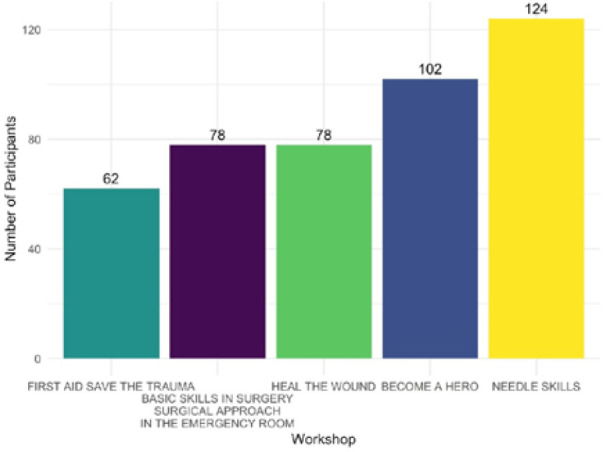
Distribution of participants across the workshops attended

Figure 3 illustrates the distribution of respondents across each workshop. Further analysis was conducted among these groups.

Regarding self-confidence, participants initially reported high levels before attending the workshops, with 50.5% assigning themselves a score of seven or higher. Following the workshops, this percentage increased to 81.7%. The specific workshop attended did not significantly influence the increase in self-confidence, as indicated by a *P* value of 0.454 (Kruskal–Wallis test). Additionally, pairwise comparisons revealed no significant differences, with all *P* values exceeding 0.1 (Mann–Whitney U test).

The transition from theoretical knowledge to practical skills was rated at seven or higher by approximately 37.6% of respondents before the workshop, increasing to 98.9% following its completion. Pairwise comparisons revealed no statistically significant differences between workshops in the distribution of responses on this topic, with all *P* values exceeding 0.1 (Mann–Whitney U test).

Regarding first aid, 63.4% of respondents considered it extremely necessary to incorporate first aid education in schools, rating it a 10 on the scale. Before participating in the workshop, 72% of the participants reported a confidence level of less than 5 in administering first aid. In contrast, 73% reported a confidence level of 10 or higher after the workshops. Pairwise comparisons between workshops revealed no statistically significant differences in the distribution of responses to these questions, with all *P* values exceeding 0.1 (Mann–Whitney U test). Participants were also asked about previous situations in which they might have had to provide first aid. Most (87.6%) reported never having been in such a situation. The remaining respondents had encountered minor incidents, primarily involving mild trauma. Two participants reported experiencing severe events: one was involved in a car crash, while another had to administer first aid to a patient undergoing an epileptic seizure.

Table 2 presents a comparative analysis of survey responses based on participants' educational status, distinguishing between those who have completed high school and those still enrolled.

**Table 2:** Comparative analysis of high school graduates and current high school students

Variable^a^		High school graduate	High school student	*P* value^*^
Involvement in the learning process	Before	7 (5 - 8)	7 (6 - 8)	0.013*
	After	9 (8 - 10)	9 (9 - 10)	0.39
Motivation to learn more		10 (6 - 10)	9 (8 - 10)	0.66
Motivation	Before	8 (7 - 9)	8 (7 - 9)	0.77
	After	10 (10 - 10)	10 (9 - 10)	0.14
Increased motivation to learn		10 (8 - 10)	10 (8 - 10)	0.57
Self-confidence	Before	7 (5 - 7.5)	6 (5 - 8)	0.68
	After	8 (7 - 9)	8 (7 - 9)	0.70
Self-trust		10 (8 - 10)	10 (8 - 10)	0.57
Theoretical knowledge	Before	6 (4.5 - 7)	6 (4 - 7)	0.64
	After	10 (9 - 10)	9 (9 - 10)	0.49
Practical abilities	Before	5 (2 - 7)	4 (3 - 6)	0.15
	After	10 (9 - 10)	10 (8 - 10)	0.64
Weekly number of hours spent studying	Before	10 (6 - 15)	10 (6.5 - 14)	0.60
	After	15 (10 - 18)	14 (11.5 - 16.5)	0.54
Correlation between practical and theory	Before	6 (4 - 7)	6 (5 - 7)	0.59
	After	9 (8 - 10)	9 (8 - 10)	0.70
Ability to administer first aid	Before	4 (2 - 5)	3 (2 - 6)	0.66
	After	8 (6 - 10)	8 (6.5 - 9)	0.22
Would you administer first aid now?		10 (5 - 10)	10 (8.5 - 10)	0.73
Do you think first aid should be taught more in schools?		10 (7 - 10)	10 (7 - 10)	0.95
Is the workshop a source of extrinsic motivation?		10 (8 - 10)	10 (8.5 - 10)	0.76
Did the workshop increase my determination to learn more?		10 (9 - 10)	10 (9 - 10)	0.11
I feel like I am preparing to become a doctor		7 (5 - 8)	7 (6 - 8)	0.01

a for each variable, the median, and IQR are presented, *Statistically significant, *P* < 0.01, Mann-Whitney U test

## DISCUSSION

The results of this study highlight that the primary motivator behind the willingness to pursue medicine was a humanitarian factor, the desire to help others (*n* = 116). The second factor was a scientific one (*n* = 50). The first two motivators are the same as in most high-income countries [18-22]. Additionally, participants cited another humanitarian reason: the desire to save lives (*n* = 14). Fascination (*n* = 6) and passion (*n* = 14) were also mentioned as scientific motivators. Although fewer participants (*n* = 70) indicated that scientific factors were most important, more participants (*n* = 130) preferred humanitarian factors. However, Wouters *et al*. [23] and Kavousipour *et al*. [24] reported that scientific factors played a greater role in their respective studies.

In our study, humanitarian motivators remained the most significant overall. Other research shows that parental influence is often an essential motivator in low- and middle-income countries [25, 26], but we found this to be minimal in high-income settings, with only one participant citing parental pressure as a key factor. One factor that has not been mentioned in any of the studies before is the emotional one. For a couple of participants (*n* = 2), the death of a parent has been the main catalyst behind their desire. This reason is probably rooted in their desire to help others, proving that the humanitarian cause remains the main one.

In most cases considered, the humanitarian factor remains the primary motivating factor. That is true for most low-middle-income countries (e.g., India) for reasons specific to lower [27] and higher standards of living. In line with previous studies in European countries such as Poland, Germany, and Hungary [5-7,28], both scientific and humanitarian factors remained influential. In contrast, research in South America (Brazil) suggests that societal factors can outweigh scientific and humanitarian motivators [29].

Regarding improvements in the before-and-after evaluations, our study revealed significant gains in all areas analyzed. In the case of motivation, there was a compelling evolution: the median score increased from 8 (IQR 7–9) before the workshop to 10 (IQR 10–10) afterward. All motivational areas analyzed showed statistically significant growth (*P* < 0.001). Although previous studies have documented increased motivation among medical students [30,31] or residents [32], our findings highlight a gap in the literature regarding the impact of such workshops on future medical students [3]. Theoretical and practical abilities proved a robust increase from 5 (IQR 3-6) when it comes to practice and growth from 6 (IQR 4-7) to 10 (IQR 9-10) in the case of theory. Practice must lead to theory, but the journey from understanding theory to applying it is challenging. Before the workshop, participants reported a correlation between practice and theory of 6 (IQR 5-7), with these results growing to 9 (IQR 8-10). The method presented in our study confirms that participants gain long-term improvement in theoretical and practical abilities [33-35]. Haupt *et al*. [36] also noted that students with limited theoretical knowledge often struggle to translate theory into practice. In our study, the workshops have been an excellent success for future medical students because of the significant increase in both the theoretical and practical parts associated with feedback on the correlation between practice and theory.

Another key aspect influencing societal benefits is first-aid training. Participants strongly supported that first aid should be taught more extensively in schools, rating its importance at a median of 10 (IQR 7–10). After the workshop, most participants reported a willingness to administer first aid, increasing their confidence from a median of 4 (IQR 2–6) to 8 (IQR 6–9). Reveruzzi *et al*. also demonstrated significant improvements in long-term self-reported first-aid knowledge and underscored the importance of integrating first-aid education in schools [37,38].

One last aspect of societal gain evaluated was the feeling of preparing to become a doctor, which increases motivation. As before, significant results were obtained with a median value of 10 (IQR 9-10) [39]. Such an increase in motivation to pursue a medical career stands as a testament to the workshop's impact. Vakayil *et al*. observed similar outcomes when assessing undergraduate students' desire to pursue surgery, although the subject focus in their study differed from ours [39]. Even though the subjects were different, the impact of practical workshops was similar, where participants showed increased motivation and societal interaction.

Participants attended between one and five workshops. Statistical analysis using the Kruskal–Wallis test (*P* = 0.454) and the Mann–Whitney U test (*P* > 0.1) revealed that the specific workshop attended did not significantly affect the increase in self-confidence. These results indicate that, regardless of the workshop subject, self-confidence is consistently improved [40-42].

Theoretical learning takes up most of the medical training. Future medical students' development can be enhanced through experiential learning, particularly hands-on workshops [43]. The advantages of experiential learning, such as increased confidence, motivation, knowledge, and effective evaluation, have been emphasized for a long time and are the same as our advantages exposed [44]. Furthermore, research consistently emphasizes that experiential learning equips students with broad knowledge and skills [45,46].

Improving the learning process, as well as the awareness of the complexity of the medical field through participation in practical workshops, represents the key to the success of future doctors. The practical applicability of already acquired theoretical notions serves as a motivation to evolve and step out of the comfort zone, thus discovering new desires, stimulating curiosity for the unknown, and achieving strong correlations between theory and practice [47,48]. Early involvement in simulation programs also promotes the development of skills and professional beliefs that align with students’ unique personalities and abilities [49]. Additionally, participating in these types of medical events provides future students with a more comprehensive understanding of their future roles in clinical health practice [50].

The skills taught in this workshop require ongoing refinement through continuous practice. A key strength of the workshop was its ability to reinforce participants' enthusiasm for medicine and encourage skill development. Additionally, it highlighted a broader societal issue—the inadequate preparation for administering first aid. While all participants were already committed to a medical career, the workshop provided valuable hands-on experience, boosting their self-confidence and readiness to engage in real-life medical situations.

To further assess the effectiveness of such interventions, we recommend conducting additional studies with a more experimental-focused design to better evaluate their impact. Moreover, expanding similar workshops to include premedical students could enhance early exposure to essential medical skills, fostering competence and confidence before entering medical training. Additionally, future initiatives should consider including participants who are not already committed to a medical career, as this could help improve first aid preparedness in the general population. Future research should explore the long-term benefits of these workshops through diverse participant groups and extended follow-up periods.

### Study limitations

This study has some limitations that must be considered when interpreting the results. First, no validated survey exists for this specific topic. Second, all participants were already committed to pursuing medical education, which may introduce selection bias. Furthermore, those who volunteered for the workshops may have already had higher baseline motivation, making it difficult to determine how much the workshops influenced their decisions. Additionally, participants were assessed approximately one month after the final workshop, leaving little time to evaluate long-term behavioral changes, such as their ability to provide first aid or maintain an increased study workload. A follow-up for 1 year could give better insights into whether the impact is sustained over time.

## CONCLUSION

This study demonstrates that humanitarian and scientific factors are the primary motivators for pursuing a medical career. Consistent with trends in other high-income countries, these factors are also key drivers for future generations of medical students in Romania and other Central-Eastern European nations. Moreover, for future medical students, participating in workshops represented an extrinsic motivational source that positively impacted motivation, self-confidence, and theoretical and practical knowledge. Finally, offering these opportunities benefits the participants and society as a whole by promoting first-aid education and encouraging the general population to engage in first-aid practices.

## Data Availability

Data and materials are available from the corresponding author upon reasonable request.
