# Supplementary material for: Impact of hands-on workshops on future medical students' motivation, confidence, and career aspirations: an observational study
Source: J Med Life. 2025 Feb;18(2):147–54. doi: 10.25122/jml-2025-0030 (PMC11932505; doi:10.25122/jml-2025-0030)
Supplement: Supplementary file 1 [file JMedLife-18-147-s001.pdf]

SECTION I

1. Surname initial + Forename initial + Age ....

2. Age ....

3. I am:
   a. High school student
   b. I graduated high school

4. In a few words, why do you want you want to pursue medicine:

5. The workshops I have participated in:
   a. Become A Hero
   b. Needle Skills
   c. Basic Skills In Surgery
   d. Surgical Approach In The Emergency Room
   e. First Aid Save The Trauma (F.a.s.t)
   f Heal The Wound

6. In a few words, why did you decide to participate in the Learn By Practice workshops?

7. On a scale from 1 to 10, what was your level of involvement in the learning process for the medical school admission before participating in the workshop/workshops? (where 1 means the minimum level of involvement and 10 represents the maximum level)

	1	2	3	4	5	6	7	8	9	10	
MINIMAL LEVEL	☐	☐	☐	☐	☐	☐	☐	☐	☐	☐	MAXIMUM LEVEL

8. On a scale from 1 to 10, what is your level of involvement in the learning process for medical school admission after participating in the workshop/workshops? (where 1 means the minimum level and 10 represents the maximum level)

	1	2	3	4	5	6	7	8	9	10	
MINIMAL LEVEL	☐	☐	☐	☐	☐	☐	☐	☐	☐	☐	MAXIMUM LEVEL

9. On a scale from 1 to 10, what was your level of desire to become a doctor before participating in the workshop/workshops?

	1	2	3	4	5	6	7	8	9	10	
MINIMAL LEVEL	☐	☐	☐	☐	☐	☐	☐	☐	☐	☐	MAXIMUM LEVEL

10. On a scale from 1 to 10, what is your desire to become a doctor after participating in the workshop/workshops?

	1	2	3	4	5	6	7	8	9	10	
MINIMAL LEVEL	☐	☐	☐	☐	☐	☐	☐	☐	☐	☐	MAXIMUM LEVEL

11. On a scale from 1 to 10, what was your level of self-confidence before participating in the workshop/workshops?

	1	2	3	4	5	6	7	8	9	10	
MINIMAL LEVEL	☐	☐	☐	☐	☐	☐	☐	☐	☐	☐	MAXIMUM LEVEL

© 2025 by the authors. This article is an open access article distributed under the terms and conditions of the Creative Commons Attribution (CC BY 4.0) license.

12. On a scale from 1 to 10, what was your theoretical knowledge level before the workshop/workshops? (about the subjects presented during the workshop)

	1	2	3	4	5	6	7	8	9	10	
MINIMAL LEVEL	☐	☐	☐	☐	☐	☐	☐	☐	☐	☐	MAXIMUM LEVEL

13. On a scale from 1 to 10, what is your theoretical knowledge level after the workshop/workshops? (about the subjects presented during the workshop)

	1	2	3	4	5	6	7	8	9	10	
MINIMAL LEVEL	☐	☐	☐	☐	☐	☐	☐	☐	☐	☐	MAXIMUM LEVEL

14. On a scale from 1 to 10, what was your practical knowledge level before the workshop/workshops? (about the subjects presented during the workshop)

	1	2	3	4	5	6	7	8	9	10	
MINIMAL LEVEL	☐	☐	☐	☐	☐	☐	☐	☐	☐	☐	MAXIMUM LEVEL

15. On a scale from 1 to 10, what is your practical knowledge level after the workshop/workshops? (about the subjects presented during the workshop)

	1	2	3	4	5	6	7	8	9	10	
MINIMAL LEVEL	☐	☐	☐	☐	☐	☐	☐	☐	☐	☐	MAXIMUM LEVEL

16. How many hours a week did you spend studying before the workshop/workshops? (if you spent more than 20 hours/week studying, then write ”More than 20 hours”)

....

17. How many hours a week do you spend studying after the workshop/workshops? (if you spend more than 20 hours/week studying, then write ”More than 20 hours”)

....

18. On a scale from 1 to 10, how would you evaluate your level of correlation between theoretical and practical information before participating in the workshop/workshops? (1 being the inability to correlate the two and 10 being the very clear correlation between the two)

	1	2	3	4	5	6	7	8	9	10	
I CAN NOT A CORRELATE THE TWO	☐	☐	☐	☐	☐	☐	☐	☐	☐	☐	I CAN SEE STRONG CORRELATION BETWEEN THE TWO

19. On a scale from 1 to 10, how would you evaluate your level of correlation between theoretical and practical information after participating in the workshop/workshops? (1 being the inability to correlate the two and 10 being the very clear correlation between the two)

	1	2	3	4	5	6	7	8	9	10	
I CAN NOT A CORRELATE THE TWO	☐	☐	☐	☐	☐	☐	☐	☐	☐	☐	I CAN SEE STRONG CORRELATION BETWEEN THE TWO

© 2025 by the authors. This article is an open access article distributed under the terms and conditions of the Creative Commons Attribution (CC BY 4.0) license.

SECTION II

(In this part of the questionnaire, you will answer only the questions that refer to workshops that you took part in)

1. After taking part in the BECOME A HERO workshop, I have learned the following abilities:
   a. The ABC Algorithm
   b. The safety of the savior
   c. The lateral safety position
   d. Cardio-pulmonary resuscitation
   e. The Heimlich maneuver

2. After taking in the NEEDLE SKILLS workshop, I have learned the following abilities:
   a. Biological samples collecting
   b. Peripheral venous catheterization
   c. Intramuscular injection
   d. Subcutaneous injection
   e. The asepsis and antisepsis norms and protocols

3. After taking part in the BASIC SKILLS IN SURGERY AND SURGICAL APPROACH IN THE EMERGENCY ROOM workshop, I have learned the following abilities:
   a. The asepsis and antisepsis norms and protocols
   b. The surgical tools
   c. Knots
   d. Sutures
   e. Cleaning and debriding an open wound

4. After taking part in the HEAL THE WOUND workshop, I have learned the following abilities:
   a. Debriding an open wound
   b. Cleaning an open wound
   c. Dressing an open wound
   d. The tetanus vaccine
   e. Loco-regional anesthesia
   f. Hemostasis using a tourniquet

5. After taking part in the FIRST AID SAVE THE TRAUMA (F.A.S.T) workshop, I have learned the following abilities:
   a. Leadership abilities
   b. Teamwork abilities
   c. Coordinating a rescue team
   d. The ability to react to the patient's behavior and interact with them
   e. The ability to be part of a rescue team

SECTION III (Questions about personal perception)

1. On a scale of 1 to 10, how sure were you feeling about administering first-aid before the workshop/workshops? (where 1 represents the absolute uncertainty and 10 represents a very high level of certainty)

	1	2	3	4	5	6	7	8	9	10	
NOT SURE AT ALL	☐	☐	☐	☐	☐	☐	☐	☐	☐	☐	VERY SURE

2. On a scale from 1 to 10, how sure are you feeling after the workshop/workshops about administering first-aid? (where 1 represents the absolute uncertainty and 10 represents a very high level of certainty)

	1	2	3	4	5	6	7	8	9	10	
NOT SURE AT ALL	☐	☐	☐	☐	☐	☐	☐	☐	☐	☐	VERY SURE

© 2025 by the authors. This article is an open access article distributed under the terms and conditions of the Creative Commons Attribution (CC BY 4.0) license.

3. Before the workshop/workshops, have you ever been in a situation where first aid was needed?
   a. No, I haven't
   b. Yes, but I did not administer first aid
   c. Yes, and I administered first aid

4. If you were in a situation to administer first aid before the workshop/workshops and you did not, what were the reasons, and what happened?

5. If you were in a situation to administer first aid before the workshop/workshops and you did so, what were the reasons, and what happened?

6. After the workshops, were you ever in a situation where first aid assistance was necessary?
   a. No, I haven't
   b. Yes, but I did not administer first aid
   c. Yes, and I administered first aid

7. If you were in a situation where first aid assistance was necessary after taking part in the workshop/workshops, and you did not administer it, what are the reasons and what happened?

8. If you were in a situation where first aid assistance was necessary after taking part in the workshop/workshops, and you did administer it, what are the reasons and what happened?

9. At this time, I would administer first aid if needed. (on a scale from 1 to 10, where 1 is total disagreement and 10 is total agreement)

	1	2	3	4	5	6	7	8	9	10	
TOTAL DISAGREEMENT	☐	☐	☐	☐	☐	☐	☐	☐	☐	☐	TOTAL AGREEMENT

10. n the following set of questions, please write the level that fits you the best (on a scale from 1 to 10, where 1 is the minimum level and 10 is the maximum)

a. The workshop represents a motivational source

	1	2	3	4	5	6	7	8	9	10	
MINIMAL LEVEL	☐	☐	☐	☐	☐	☐	☐	☐	☐	☐	MAXIMUM LEVEL

© 2025 by the authors. This article is an open access article distributed under the terms and conditions of the Creative Commons Attribution (CC BY 4.0) license.

b. The workshop gave me the confidence to administer first aid if needed

	1	2	3	4	5	6	7	8	9	10	
MINIMAL LEVEL	☐	☐	☐	☐	☐	☐	☐	☐	☐	☐	MAXIMUM LEVEL

c. The workshop made me more determined to be more involved in the studies necessary for my medical school admission exam

	1	2	3	4	5	6	7	8	9	10	
MINIMAL LEVEL	☐	☐	☐	☐	☐	☐	☐	☐	☐	☐	MAXIMUM LEVEL

d. The workshop made me more determined

	1	2	3	4	5	6	7	8	9	10	
MINIMAL LEVEL	☐	☐	☐	☐	☐	☐	☐	☐	☐	☐	MAXIMUM LEVEL

e. The workshop made me feel as if I was preparing to become a doctor

	1	2	3	4	5	6	7	8	9	10	
MINIMAL LEVEL	☐	☐	☐	☐	☐	☐	☐	☐	☐	☐	MAXIMUM LEVEL

f. The workshop made me study more

	1	2	3	4	5	6	7	8	9	10	
MINIMAL LEVEL	☐	☐	☐	☐	☐	☐	☐	☐	☐	☐	MAXIMUM LEVEL

g. I feel that first aid administration is not taught enough in schools

	1	2	3	4	5	6	7	8	9	10	
MINIMAL LEVEL	☐	☐	☐	☐	☐	☐	☐	☐	☐	☐	MAXIMUM LEVEL

© 2025 by the authors. This article is an open access article distributed under the terms and conditions of the Creative Commons Attribution (CC BY 4.0) license.
